# Prospective telehealth analysis of functional performance, frailty, quality of life, and mental health after COVID-19 hospitalization

**DOI:** 10.1186/s12877-022-02854-6

**Published:** 2022-03-26

**Authors:** Jacob J. Capin, Melissa P. Wilson, Kristine Hare, Swati Vempati, Carley E. Little, Donna McGregor, Jose Castillo-Mancilla, Jennifer E. Stevens-Lapsley, Sarah E. Jolley, Kristine M. Erlandson

**Affiliations:** 1 Physical Therapy Program, Department of Physical Medicine and Rehabilitation, University of Colorado Anschutz Medical Campus, Aurora, CO USA; 2 Department of Physical Therapy, Marquette University, Milwaukee, WI USA; 3 Eastern Colorado Veterans Affairs Geriatric Research Education and Clinical Center (GRECC), Aurora, CO USA; 4 Department of Bioinformatics and Personalized Medicine, University of Colorado Anschutz Medical Campus, Aurora, CO USA; 5 Division of Infectious Diseases, University of Colorado Anschutz Medical Campus, 12700 E. 19th Ave, Mail Stop B168, Aurora, CO 80045 USA; 6 School of Medicine, University of Colorado Anschutz Medical Campus, Aurora, CO USA; 7 Division of Pulmonary and Critical Care Medicine, University of Colorado Anschutz Medical Campus, Aurora, CO USA

**Keywords:** COVID19, Frailty, Function, Quality of life, Posttraumatic stress disorder, Recovery

## Abstract

**Background:**

COVID-19 is a global pandemic with poorly understood long-term consequences. Determining the trajectory of recovery following COVID-19 hospitalization is critical for prioritizing care, allocating resources, facilitating prognosis, and informing rehabilitation. The purpose of this study was to prospectively evaluate recovery following COVID-19 hospitalization.

**Methods:**

Participants age 18 years or older who were hospitalized for ≥24 h due to COVID-19 completed phone/video call virtual assessments (including the 10-time chair rise test) and survey forms at three time points (2–6, 12, and 18 weeks) after hospital discharge. Univariate logistic and linear regression models assessed the associations of the outcomes with primary predictors (categorical age, sex, race/ethnicity group, and categorical pre-hospitalization frailty) at baseline; the same were used to assess differences in change from week 2–6 (continuous outcomes) or outcome persistence/worsening (categorical) at last contact.

**Results:**

One hundred nine adults (age 53.0 [standard deviation 13.1]; 53% female) participated including 43 (39%) age 60 or greater; 59% identified as an ethnic and/or racial minority. Over 18 weeks, the mean time to complete the 10-time chair rise test decreased (i.e., improved) by 6.0 s (95% CI: 4.1, 7.9 s; *p* < 0.001); this change did not differ by pre-hospital frailty, race/ethnicity group, or sex, but those age ≥ 60 had greater improvement. At weeks 2–6, 67% of participants reported a worse Clinical Frailty Scale category compared to their pre-hospitalization level, whereas 42% reported a worse frailty score at 18 weeks. Participants who did not return to pre-hospitalization levels were more likely to be female, younger, and report a pre-hospitalization category of ‘very fit’ or ‘well’.

**Conclusions:**

We found that functional performance improved from weeks 2–6 to 18 weeks of follow-up; that incident clinical frailty developed in some individuals following COVID-19; and that age, sex, race/ethnicity, and pre-hospitalization frailty status may impact recovery from COVID-19. Notably, individuals age 60 and older were more likely than those under age 45 years to return to their pre-hospitalization status and to make greater improvements in functional performance. The results of the present study provide insight into the trajectory of recovery among a representative cohort of individuals hospitalized due to COVID-19.

**Supplementary Information:**

The online version contains supplementary material available at 10.1186/s12877-022-02854-6.

## Background

Coronavirus disease (COVID)-19 is a global pandemic with poorly understood long-term consequences. Recent data suggest that even mild cases of COVID-19 can result in significant long-term morbidity [1]. Determining the trajectory of recovery in patients following COVID-19 hospitalization is critical for prioritizing care, allocating resources, facilitating prognosis, and informing rehabilitation.

Post-acute sequelae of COVID-19 (PASC), often defined as symptoms persisting at least 4 weeks beyond initial symptom onset, may affect many organ systems including pulmonary, hematologic, cardiovascular, neuropsychiatric, renal, endocrine, gastrointestinal, hepatobiliary, and dermatologic [2]. Sixty days after hospital discharge, cardiopulmonary symptoms such as cough and dyspnea, difficulty completing activities of daily living, inability to return fully to work, and emotional disturbances are among the most commonly reported symptoms [3]. Six months after hospital discharge, the most common symptoms reported by 1733 COVID-19 survivors from Wuhan, China were fatigue or muscle weakness (63%), sleep difficulties (26%), and anxiety and/or depression (23%); nearly one-quarter had 6-min walk test (6-MWT) values below the lower limit of the normal range [4]. These studies suggest a high prevalence of symptoms and functional limitations persist for weeks to months in patients after COVID-19 hospitalization.

Although the aforementioned studies and others [2–8] have begun to inform clinical practice and prognosis for patients with post-acute sequelae of COVID-19 (PASC), limitations to our understanding of the sequelae of COVID-19 remain. Most studies to date are limited to one cross-sectional evaluation [2–7], rather than longitudinal follow-up. Several studies have been performed at post-COVID clinics [5, 7], in rehabilitation units [9], or through social media outreach of COVID-19 support groups [10], which could bias towards individuals with persistent or more severe symptoms. Another limitation of prior work is a dearth of performance-based functional assessments, as many studies rely heavily or exclusively on chart review and/or patient-reported outcomes [3, 6, 7]. Similarly, while many studies have identified frailty as a strong predictor of COVID-19 disease severity and mortality [11–15], the effect of COVID-19 hospitalization on the development and/or progression of clinical frailty is unknown. These limitations point to a need for prospective, serial evaluation of outcomes, including performance-based functional assessments, in a representative sample of ‘all-comers’ following COVID-19 hospitalization.

The purpose of this study was to prospectively evaluate and describe recovery in patients at multiple time-points following a hospitalization due to COVID-19. We hypothesized that: 1) outcomes would improve over time (from 2 to 6 weeks post hospital discharge to 12 and 18 weeks post-discharge); 2) COVID-19 hospitalization would result in clinical frailty in some patients who did not report pre-hospitalization frailty; and 3) age, sex, race/ethnicity, and pre-hospitalization status would impact recovery from COVID-19. These a priori hypotheses were guided by: 1) the natural course of recovery experienced by most individuals following many acute illnesses; 2) the prevalence of PASC and nosocomial-related functional decline; and 3) prior studies identifying associations between patient characteristics and outcomes following COVID-19 and other diseases.

## Methods

### Design

Prospective cohort study.

### Ethics and consent to participate

All methods were carried out in accordance with the Declaration of Helsinki, and the study was approved by the Colorado Multiple Institutional Review Board (COMIRB 20–0703 and COMIRB 20–0690). All participants provided electronic informed consent (via REDCap).

### Setting and recruitment

Potential participants were identified using hospital discharge records at the University of Colorado Hospital (Aurora, CO). Individuals were eligible to participate in this study if they were at least 18 years of age and had been hospitalized due to COVID-19 for more than 24 h between March 2020 and November 2020 and subsequently discharged home (or prior place of residence); participants requiring a higher level of care (e.g., skilled nursing facility) were not included. Participants were required to speak and read either English or Spanish, be able to provide informed consent, and be able to access online questionnaires through a computer, tablet, or smart phone. While individuals could be enrolled in the study up to 18 weeks following hospital discharge, most were contacted and enrolled within the first 6 weeks. Supplemental Fig. 1 provides a flow chart of screening and enrollment.

### Characteristics of participants

Characteristics of participants and of their hospitalizations were collected from medical record abstraction. Data gleaned from the medical record included duration of hospitalization, need for and duration of mechanical ventilation, need for and duration of intensive care unit (ICU) stay, comorbidities, discharge location, and need for supplemental oxygen at discharge.

### Virtual (video or phone) visits

Participants completed virtual research visits by video (study preference) or telephone at 2–6 weeks, 12 weeks, and 18 weeks post-hospital discharge. Participants were asked about changes in or lack of symptoms including fever, shortness of breath, cough, dizziness, confusion, fatigue, anosmia (i.e., loss of smell), and ageusia (i.e., loss of taste).

### Functional performance assessment (10-time chair rise test)

The 10-time chair rise test is a component of the expanded Short Physical Performance Battery (SPPB), a validated assessment of hospitalization, morbidity, and mortality in middle-aged and older populations [16–18], to capture function in higher-performing adults [19]. The test is performed using a chair with no arms or padding approximately 45 cm in height (i.e., a standard kitchen table chair). For this study, testing was observed via video or, to facilitate technological challenges and allow for telephone collection for those unable or unwilling to use video, participants counted out loud as they stood from the chair while on speaker phone. The same method (video versus telephone) was used for each follow-up assessment. Participants were first asked to rise from the chair once. If the participant was unable to rise from the chair or did not feel comfortable standing, the test was stopped; otherwise, the person was subsequently asked to rise 10 times as quickly as safely possible. Chair rise time is responsive to change and predictive of outcomes [20, 21].

### Patient reported outcome measures

Participants completed patient reported outcome measures via online questionnaires on REDCap and/or telephone calls during the post-hospital assessment period. All surveys were available in English and Spanish and distributed to the participant in his or her preferred language.

#### Clinical frailty scale

Participants estimated their function pre- and post-hospitalization using the Rockwood Clinical Frailty Scale [22], an easy to interpret estimate of function than has been recently applied extensively to participants with COVID-19 [11–15, 23]. Participants estimated pre-hospitalization frailty level at their first post-discharge follow-up assessment (typically 2–6 weeks post-discharge), and current frailty at each assessment. The scale consists of nine ordinal responses ranging from (1) very fit to (9) terminally ill. We categorized participants with scores of 1 (very fit) and 2 (well) as non-frail and participants with scores of 3 (managing well) or worse as pre-frail/frail. The Clinical Frailty Scale was administered online via REDCap.

#### MRC dyspnea scale

The MRC Dyspnea Scale assesses perceived difficulty breathing by answering yes or no to five statements from, ‘I only get breathless with strenuous exercise’ to ‘I am too breathless to leave the house’ [24]. The MRC Dyspnea Scale was administered during the phone/video call.

#### The World Health Organization disability assessment schedule (WHODAS 2.0)

The World Health Organization Disability Assessment Schedule 2.0 short form (WHODAS 2.0) measures disability due to health conditions including diseases, illness, injuries, mental or emotional problems, and problems with alcohol or drugs. The 12-item questionnaire covers six different adult life tasks including understanding and communication, self-care, mobility, interpersonal relationships, work and household activities, and community and civic roles. The sum score for global disability ranges from 0 (no disability) to 48 (complete disability) with higher scores representing greater (worse) disability. The WHODAS 2.0 has published normative data [25] and was administered via REDCap.

#### Impact of event scale-revised (IES-6)

The Impact of Event Scale-Revised (IES-6) is an abbreviated six-item self-report measure that assesses subjective distress caused by traumatic events [26] and has been validated in survivors of acute respiratory distress syndrome [27]. Respondents indicate how much they were distressed or bothered during the past seven days, as a consequence of a recent event (i.e., hospitalization due to COVID-19). The IES-6 was administered via REDCap.

### Statistical analysis

Race and ethnicity information were combined to create four participant sub-groups: 1) White, non-Hispanic (reference group); 2) Black, non-Hispanic; 3) Hispanic (regardless of race); 4) and Other/Unknown. Due to small sample size (*n* = 6), the “Other/Unknown” group was excluded from modeling. Age was categorized into: 1) age < 45 years (reference group), 2) age 45–59 years, and 3) age ≥ 60 years. Pre-hospitalization frailty was dichotomized as 1) non-frail and 2) pre-frail/frail according to the previously described Clinical Frailty Score categories. Linear regression (10-time chair rise test, WHODAS 2.0) or logistic regression (categorical outcomes) models were used to assess relationships between outcomes and primary predictors at baseline; the same were used to explore differences in improvement from week 2–6 (continuous outcomes) or persistence/worsening of outcome measure (categorical outcomes). Results were interpreted as significant in the context of the *p*-value (alpha = 0.05), estimate (odds ratio [OR], or units specified), and 95% confidence interval (CI). No adjustments were made for multiple comparisons [28]. All graphic creation and data analysis was performed using R statistical software (version 4.1.1).

## Results

### Participants and participant characteristics

**Fig. 1:**
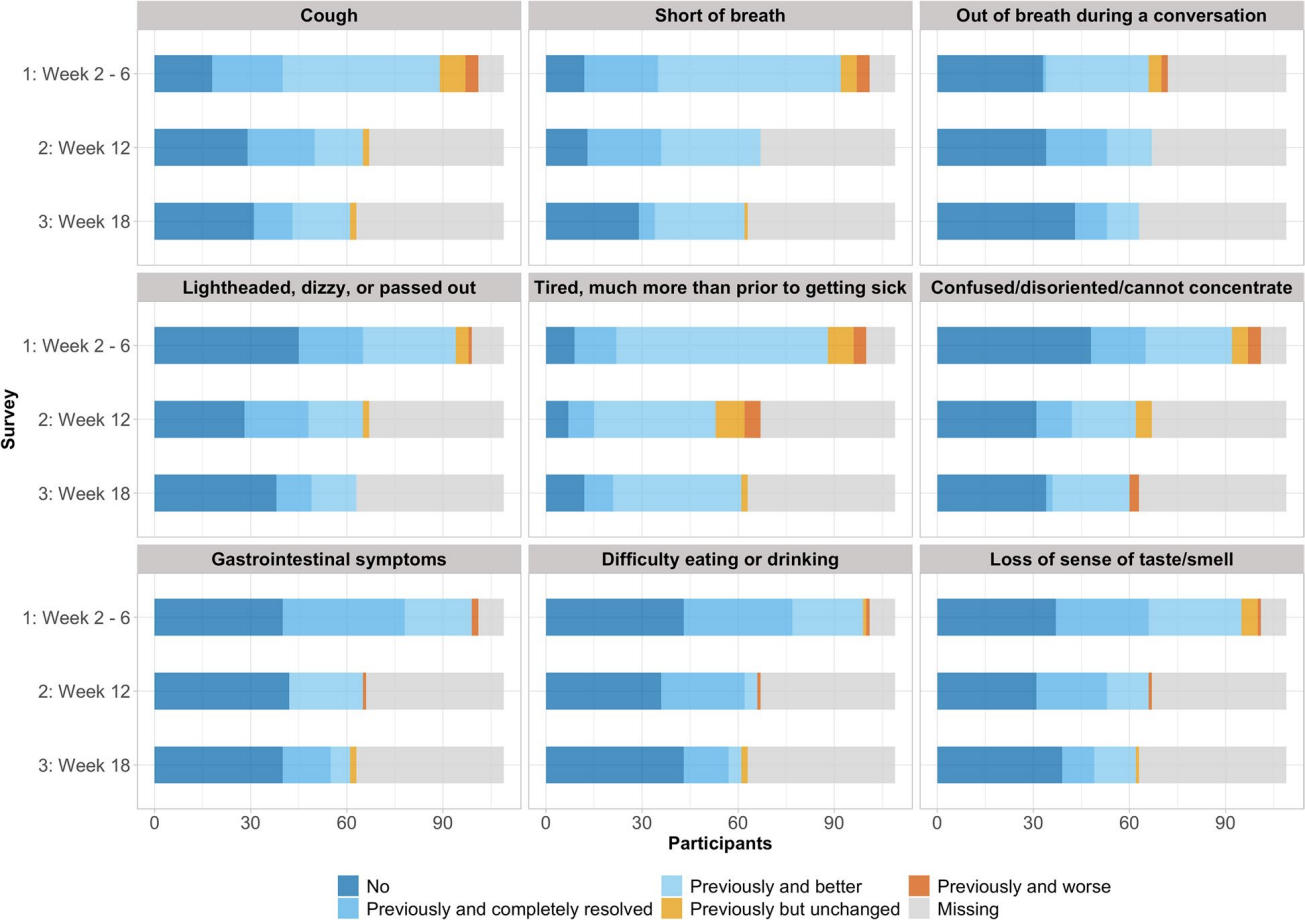
Participant-reported symptoms by survey week

One hundred nine individuals, including 43 (39%) age 60 and older, participated in the study (Table 1). Categorical age distribution differed by race/ethnicity group (*p* = 0.001), with Hispanic participants tending to be < 60 years of age. Detailed characteristics classified by participant age (Additional file 1: Appendix 1), sex (Additional file 1: Appendix 2), and race/ethnicity groups (Additional file 1: Appendix 3) may be found in the appendices. Longitudinal changes in post-hospital COVID-19 symptomatology at each assessed time point are shown in Fig. 1.

**Table 1:** Demographic, social and medical characteristics

Demographics	*N* = 109
Mean age at admission (SD)	53.0 (13.1)
Male (%)	51 (46.8)
Race/Ethnicity (%)	
Black/African-American, non-Hispanic	24 (22.0)
Hispanic, regardless of race	34 (31.2)
Other race, non-Hispanic	6 (5.5)
White, non-Hispanic	45 (41.3)
Education (%)	
Some high school	5 (4.6)
High school graduate or GED	12 (11.0)
Some college or associate’s degree	23 (21.1)
Completed college or bachelor’s degree	14 (12.8)
Post-college (regardless of degree)	13 (11.9)
Missing	42 (38.5)
Alcohol consumption (%)	
Yes	38 (34.9)
No	57 (52.3)
Unknown	14 (12.8)
Smoker (%)	
Yes	29 (26.6)
No	74 (67.9)
Unknown	6 (5.5)
Marijuana use (%)	
Yes	8 (7.3)
No	62 (56.9)
Unknown	39 (35.8)
Substance abuse (%)	3 (2.8)
**Comorbidities**	
Cardiovascular disease (%)	15 (13.8)
Respiratory disease (%)	27 (24.8)
Renal disease (%)	10 (9.2)
Hypertension (%)	49 (45.0)
Type 2 diabetes (%)	37 (33.9)
Morbid obesity (%)	15 (13.8)
**Hospital Course**	
Median days hospitalized [IQR]	4.0 [3.0, 9.0]
Admission to intensive care unit (ICU) (%)	28 (25.7)
Intubated (%)	14 (12.8)
Discharge location (%)	
Home	98 (89.9)
Home with home health	4 (3.7)
Facility	4 (3.4)
Other	3 (2.8)
Discharged on oxygen (%)	56 (51.4)

### Intensive care unit (ICU) admission

ICU admission occurred in 26% (28/109) of participants and differed by categorical age (*p* = 0.02). Participants age 60 and older were 76% *less* likely than participants under 45 years to be admitted to the ICU (odds ratio [OR]: 0.2 [95% CI: 0.1, 0.7], *p* = 0.010). Participants age 45–59 years were 66% *less* likely than those under 45 years to be admitted to the ICU (OR: 0.3 [95% CI: 0.1, 1.0], *p* = 0.048). ICU admission status did not differ by race/ethnicity, sex, or pre-hospitalization frailty category in our sample of post-hospital participants (*p* > 0.3).

### Assessment completion

A total of 247 phone/video call assessments (symptoms, 10-time chair rise test, MRC Dyspnea) and 221 survey forms (Clinical Frailty Scale, activity level, WHODAS 2.0, IES-6) were completed over the 3 time points (Additional file 1: Appendix 4). All 109 participants completed at least one call and 78% (85/109) of participants completed surveys at one or more time point. Participants who only participated at a single time point (*n* = 29) were more likely to be younger (mean age: 48.5 vs 54.6 years; *p* = 0.034), less likely to identify as non-Hispanic, White (21% vs 49%; *p* = 0.016), and less likely to have completed any survey (48% vs 89%; *p* < 0.001), as compared to participants who had data collected at multiple time points.

#### Functional performance

##### 10-time chair rise test

At the week 2–6 visit, 72% (73/101) of participants completed the 10-time chair rise test (Table 2). Those who could versus could not complete the exercise did not differ by sex, categorical age, or race/ethnicity (Tables 3, 4, 5 and 6). Females took longer than males (Table 3) and both African American and Hispanic participants took longer than non-Hispanic White participants (Table 4) to complete the 10-time chair rise test.

**Table 2:** Responses to primary and secondary outcomes measures at 2–6, 12, and 18 weeks post-discharge are provided

	**Weeks 2–6**	**Week 12**	**Week 18**
**Interview Calls Completed**	***N*** **= 101**	***N*** **= 67**	***N*** **= 63**
**10-Time Chair Rise Test**^a^			
Mean [95% CI] seconds; N	29 [26, 32]; *N* = 73	25 [22, 28]; *N* = 50	21 [19, 23]; *N* = 43
Unable to complete due to physical ability or safety concerns, % (n/total)	16% (16/101)	12% (8/67)	11% (7/63)
Refused test (inconvenient), % (n/total)	12% (12/101)	13% (9/67)	21% (13/63)
**MRC Dyspnea**			
Dyspnea only with strenuous exercise or when hurrying, % (n/total)	38% (38/101)	64% (43/67)	71% (45/63)
Walking slower than most people or stopping on level ground due to dyspnea, % (n/total)	37% (37/101)	21% (14/67)	17% (11/63)
Dyspnea upon dressing and/or dyspnea that prevented them from leaving the house, % (n/total)	26% (26/101)	15% (10/67)	11% (7/63)
**Supplemental O2 Use**			
% (n/total)	40% (40/101)	27% (18/67)	29% (18/63)
**Therapy**			
In-home care, % (n/total)	3% (3/101)	3% (2/67)	0% (0/63)
Outpatient PT and/or OT, % (n/total)	10% (10/101)	3% (2/67)	11% (7/63)
	**Weeks 2–6**	**Week 12**	**Week 18**
**Survey Form (one or more section completed)**	***N*** **= 69**	***N*** **= 56**	***N*** **= 59**
**Reported Clinical Frailty Scale score**^a^			
Pre-frail/frail score, % (n/total)	77% (51/66)	59% (33/56)	51% (30/59)
Return to pre-hospitalized level, % (n/total)	33% (21/64)	55% (27/49)	60% (29/48)
**IES-6**			
Probable PTSD, % (n/total)	45% (31/69)	20% (11/56)	22% (13/59)
No PTSD, % (n/total)	55% (38/69)	80% (45/56)	78% (46/59)
**WHODAS 2.0**			
Mean [95% CI]; N	15 [12, 17]; *N* = 66	9 [6, 11]; *N* = 56	7 [5, 9]; *N* = 59
**Reported exercise**			
Return to pre-hospitalization level, % (n/total)	(Unavailable)^b^	63% (29/46)	69% (31/45)

^a^Primary outcome; ^b^data collected at weeks 12 and 18 only

**Table 3:** Differences in impairment among females compared to males, presented as estimate or odds ratio (OR) when indicated with (95% confidence interval) and *p*-values for each outcome. Estimates that are statistically significant (*p* < 0.05) are bolded

Outcome; estimate type	Baseline difference Est [95% CI] *P*-value	Week 18 difference^*^ Est [95% CI] *P*-value
**10-Time Chair Rise Test**		
Unable to complete due to physical ability or safety concerns; OR	2.1 [0.7, 7.4] *P* = 0.20	
Time to complete; seconds	**5.6 [0.2, 11.1]** ***P*** **= 0.04**	
Change in time to complete from week 2/6; seconds		0 [−3.2, 4.1] *P* = 0.81
**Clinical Frailty Scale**		
Post-hospitalization reported frailty; OR	0.8 [0.2, 2.5] *P* = 0.67	
Did not return to pre-hospitalization frailty; OR		**3.4 [1.2, 10.6]** ***P*** **= 0.03**
**MRC Dyspnea Scale**		
Too SOB to leave the house and/or SOB while dressing; OR	2.0 [0.8, 5.3] *P* = 0.13	
No improvement from week 2–6 answer; OR		1.6 [0.6, 4.9] *P* = 0.37
**IES-6**		
Met criteria for PTSD; OR	1.3 [0.5, 3.5] *P* = 0.53	
Continued or new PTSD; OR		2.7 [0.9, 9.6] *P* = 0.10
**WHODAS 2.0**		
Total score	4.4 [−0.3, 9.1] *P* = 0.06	
Difference from week 2–6; score		3.1 [−1.8, 8.1] *P* = 0.21
**Supplemental O2**		
Reported use; OR	2.0 [0.9, 4.5] *P* = 0.10	
Continued or new use of supplemental O2; OR		**4.8 [1.5, 19.0]** ***P*** **= 0.01**
**Activity Level**		
Did not return to pre-hospitalization exercise; OR		1.2 [0.4, 3.8] *P* = 0.8

^*^ Week 12 data used if week 18 data was unavailable

**Table 4:** Differences in impairment for racial/ethnic minorities compared to White, non-Hispanic participants, presented as estimate or odds ratio (OR) when indicated with (95% confidence interval) and *p*-values for each outcome. Estimates that are statistically significant (*p* < 0.05) are bolded

Outcome; estimate type	Baseline difference Est [95% CI] *P*-value		Week 18 difference ^*^ Est [95% CI] *P*-value	
Race/Ethnicity category	Black/African American, NH	Hispanic, regardless of race	Black/African American, NH	Hispanic, regardless of race
**10-Time Chair Rise Test**				
Unable to complete due to physical ability or safety concerns; OR	1.2 [0.3, 4.2] *P* = 0.81	0.3 [0, 1.2] *P* = 0.13		
Time to complete; seconds	**7.7 [2.0, 13.5]** ***P*** **= 0.01**	**6.0 [1.1, 11.0]** ***P*** **= 0.02**		
Change in time to complete from week 2/6; seconds			3.0 [−2.2, 8.1] *P* = 0.25	2.7 [−1.6, 7.0] *P* = 0.22
**Clinical Frailty Scale**				
Post-hospitalization reported frailty; OR	0.3 [0.1, 1.4] *P* = 0.14	0.3 [0.1, 1.2] *P* = 0.09		
Did not return to pre-hospitalization frailty; OR			0.6 [0.1, 2.2] *P* = 0.41	1.1 [0.3, 4.7] *P* = 0.87
**MRC Dyspnea Scale**				
Too SOB to leave the house and/or SOB while dressing; OR	1.1 [0.4, 3.4] *P* = 0.80	0.4 [0.1, 1.3] *P* = 0.14		
No improvement from week 2–6 answer; OR			2.9 [0.7, 12.0] *P* = 0.14	3.4 [0.9, 13.2] *P* = 0.07
**IES-6**				
Met criteria for PTSD; OR	2.8 [0.8, 10.1] *P* = 0.11	1.7 [0.5, 5.7] *P* = 0.41		
Continued or new PTSD; OR			3.0 [0.7, 12.3] *P* = 0.13	2.4 [0.6, 9.7] *P* = 0.21
**WHODAS 2.0**				
Total score	3.2 [−2.9, 9.3] *P* = 0.30	−4.0 [−10.0, 2.1] *P* = 0.20		
Difference from week 2–6; score			−4.1 [−10.5, 2.3] *P* = 0.20	−4.7 [− 11.5, 2.1] *P* = 0.17
**Supplemental O2**				
Reported use; OR	1.0 [0.3, 2.9] *P* = 0.99	1.5 [0.6, 3.8] *P* = 0.42		
Continued or new use of supplemental O2; OR			1.0 [0.2, 4.6] *P* = 0.96	1.9 [0.5, 6.7] *P* = 0.35
**Activity Level**				
Did not return to pre-hospitalization exercise; OR			0.9 [0.2, 3.6] *P* = 0.89	0.3 [0, 1.7] *P* = 0.23

^a^ Week 12 data used if week 18 data was unavailable

**Table 5:** Differences in impairment among participants by age category compared to participants < 45 years of age, presented as estimate or odds ratio (OR) when indicated with (95% confidence interval) and *p*-values for each outcome. Estimates that are statistically significant (*p* < 0.05) are bolded

Outcome; estimate type	Baseline difference Est [95% CI] *P*-value		Week 18 difference^*^ Est [95% CI] *P*-value	
Age category	45–59	60 or greater	45–59	60 or greater
**10-Time Chair Rise Test**				
Unable to complete due to physical ability or safety concerns; OR	1.5 [0.3, 11.3] *P* = 0.68	3.4 [0.8, 23.7] *P* = 0.14		
Time to complete; seconds	−2.3 [−9.5, 4.9] *P* = 0.52	−3.3 [−10.4, 3.8] *P* = 0.36		
Change in time to complete from week 2/6; seconds			−2.7 [−7.9, 2.6] *P* = 0.31	−5.1 [−10.3, 0] *P* = 0.05
**Clinical Frailty Scale**				
Post-hospitalization reported frailty; OR	3.2 [0.7, 18.0] *P* = 0.15	2.5 [0.6, 9.9] *P* = 0.18		
Did not return to pre-hospitalization frailty; OR			0.3 [0.1, 1.3] *P* = 0.11	0.3 [0.8, 1.1] *P* = 0.09
**MRC Dyspnea Scale**				
Too SOB to leave the house and/or SOB while dressing; OR	0.6 [0.2, 2.5] *P* = 0.48	2.4 [0.8, 8.5] *P* = 0.14		
No improvement from week 2–6 answer; OR			0.7 [0.2, 3.2] *P* = 0.65	0.3 [0.1, 1.2] *P* = 0.09
**IES-6**				
Met criteria for PTSD; OR	1.5 [0.4, 5.8] *P* = 0.51	1.6 [0.5, 5.4] *P* = 0.43		
Continued or new PTSD; OR			0.3 [0.1, 1.2] *P* = 0.10	0.3 [0.1, 1.0] *P* = 0.05
**WHODAS 2.0**				
Total score	−1.1 [−7.5, 5.2] *P* = 0.72	3.4 [−2.4, 9.2] *P* = 0.25		
Difference from week 2–6; score			0.2 [−6.8, 7.1] *P* = 0.96	2.3 [−4.2, 8.8] *P* = 0.47
**Supplemental O2**				
Reported use; OR	0.9 [0.3, 2.8] *P* = 0.91	1.3 [0.5, 3.7] *P* = 0.61		
Continued or new use of supplemental O2; OR			1.7 [0.3, 13.3] *P* = 0.58	2.8 [0.6, 20.0] *P* = 0.24
**Activity Level**				
Did not return to pre-hospitalization exercise; OR			0.3 [0.1, 1.7] *P* = 0.19	0.3 [0.1, 1.1] *P* = 0.07

^*^ Week 12 data used if week 18 data was unavailable

**Table 6:** Differences in impairment by pre-hospital pre-frail/frail compared to non-frail, presented as estimate (95% confidence intervals) and *p*-values for each outcome. Estimates that are statistically significant (p < 0.05) are bolded

Outcome; estimate type	Baseline difference Est [95% CI] *P*-value	Week 18 difference^*^ Est [95% CI] *P*-value
**10-Time Chair Rise Test**		
Unable to complete due to physical ability or safety concerns; OR	1.5 [0.3, 6.4] *P* = 0.58	
Time to complete; seconds	−0.5 [−7.9, 7.0] *P* = 0.90	
Change in time to complete from week 2/6; seconds		1.5 [−4.1, 7.1] *P* = 0.59
**Clinical Frailty Scale**		
Post-hospitalization reported frailty; OR	1.2 [0.4, 4.2] *P* = 0.81	
Did not return to pre-hospitalization frailty; OR		**0.0 [0.0, 0.2]** ***P*** **= 0.003**
**MRC Dyspnea Scale**		
Too SOB to leave the house and/or SOB while dressing; OR	1.7 [0.5, 5.1] *P* = 0.38	
No improvement from week 2–6 answer; OR		1.0 [0.3, 3.7] *P* = 0.99
**IES-6**		
Met criteria for PTSD; OR	0.6 [0.2, 1.6] *P* = 0.28	
Continued or new PTSD; OR		0.7 [0.1, 2.8] *P* = 0.64
**WHODAS 2.0**		
Total score	−0.5 [−5.4, 4.4] *P* = 0.84	
Difference from week 2–6; score		−2.2 [−7.8, 3.5] *P* = 0.45
**Supplemental O2**		
Reported use; OR	1.6 [0.6, 4.5] *P* = 0.56	
Continued or new use of supplemental O2; OR		**5.6 [1.4, 23.7]** ***P*** = **0.02**
**Activity Level**		
Did not return to pre-hospitalization exercise; OR		**0.2 [0.0, 0.8]** ***P*** **= 0.048**

^*^ Week 12 data used if week 18 data was unavailable

Among the 44 participants with 10-time chair rise test at more than one time point, 89% (39/44) improved, whereas 11% (5/44) had the same or worse time. On average, participants had a decrease (i.e., improvement) of 6.0 s (95% CI: 4.1, 7.9 s; *p* < 0.001). Those aged 60 or older tended (*p* = 0.05) to improve more than those under age 45 years (Table 5).

#### Patient reported outcome measures

##### Clinical frailty scale

**Fig. 2:**
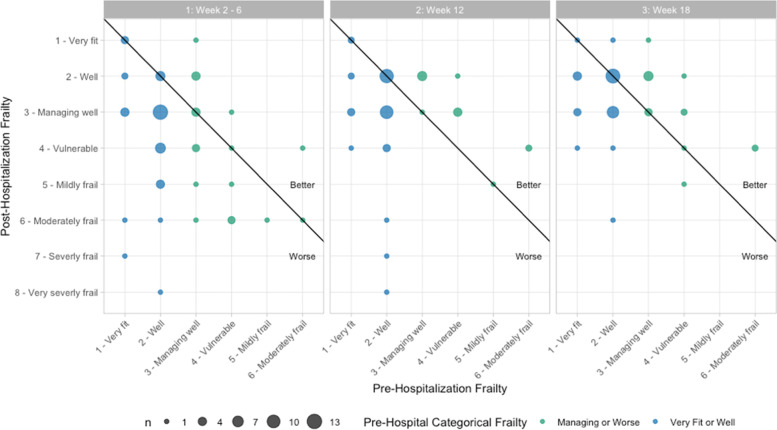
Self-reported post-hospitalization frailty compared to pre-hospitalization frailty by time point. The reference line indicates agreement between pre- and post- hospital assessment: points above the line indicate post-hospital assessment was better than pre-hospital assessment whereas points below the line indicate post-hospital assessment was worse

Seventy participants reported pre-hospitalization frailty levels, with 66% (46/70) self-identifying as non-frail (i.e., ‘very fit’ or ‘well’). At weeks 2–6, only 33% (21/64) of respondents had returned to their pre-hospitalization frailty classification (Fig. 2; Table 2). Post-hospitalization frailty (categorical) at weeks 2–6 did not differ by sex, categorical age, or race/ethnicity.

Of the 46 participants who identified as non-frail prior to their COVID-19-hospitalization, 83% (38/46) reported Clinical Frailty Scale scores consistent with new pre-frailty or frailty at one or more time point post-hospitalization. Of 59 participants who reported frailty information at week 18 (or week 12 if missing), 22% (13/59) improved, 36% (21/59) were unchanged, and 42% (25/59) reported worse frailty scores as compared to their reported pre-hospitalization baseline. Participants who did not return to their pre-hospitalization state were 3.4 times more likely to be female (Table 3), were more likely to report being well or very-well prior to their hospitalization (Table 6), and tended to be younger (Table 5).

### Secondary outcome measures

**Fig. 3:**
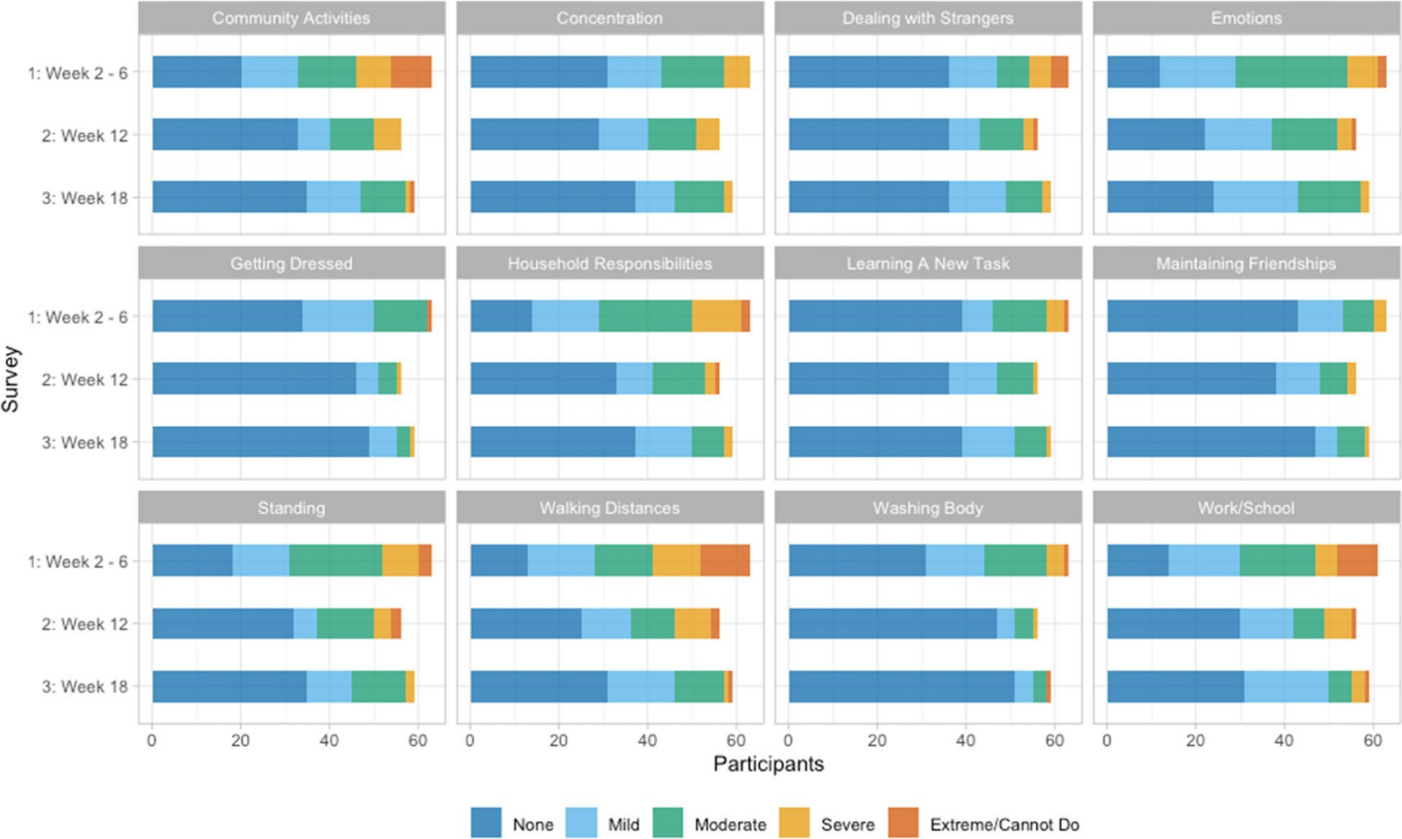
WHODAS 2.0 questionnaire results by individual question topic and survey week. Each box represents a question assessing difficulties experienced in performing the noted activity in the previous 14 days due to health-related conditions

Secondary outcome measures (Table 2), including the MRC Dyspnea Scale, WHODAS 2.0 (Fig. 3), and IES-6, were reported and interpreted in the context of the primary results. Females were 4.8 times more likely than males to report continued use of oxygen and report continued or new probable PTSD at final follow-up; odds ratios for other outcomes followed these trends, albeit less strongly (Table 3). There were no statistically significant differences in secondary outcome measures according to racial/ethnic minority group status (Table 4). Similar to the findings for the 10-time chair rise, participants aged 60 or older (compared to those under age 45) tended to be more likely to improve on the MRC Dyspnea Scale, less likely to report continued or new probable PTSD, and more likely to return to their pre-hospitalization exercise level (Table 5). Participants who were non-frail pre-hospitalization were less likely to return to their pre-hospitalization exercise level (Table 6).

## Discussion

Among a diverse group of hospitalized individuals with COVID-19, we provide a detailed assessment of post-hospital recovery, incorporating both functional performance and patient reported outcomes over 18 weeks. As we hypothesized, 10-time chair rise improved from 2-6 weeks to 18 weeks among the vast majority (89%) of participants, with an average decrease of 6.0 s, a robust and likely clinically meaningful change consistent with the effects seen after 24 weeks of supervised exercise [29]. Next, 67% of participants reported worse scores on the Clinical Frailty Scale 2–6 weeks post-discharge compared to their pre-hospitalization status. Lastly, we found that age, sex, race/ethnicity, and pre-hospitalization status impacted recovery from COVID-19, though with unique contributions to each outcome. Notably, the study sample was relatively young (mean age 53 years) and limited to individuals discharged home (or prior place of residence). Recovery may be slower among older adults with greater frailty and comorbidities who often experience more severe initial infections. Despite these limitations to generalizability, the results of the present study provide insight into the trajectory of recovery among an observational cohort of individuals who were hospitalized due to COVID-19 and may inform how providers counsel patients and/or direct additional treatments or therapies.

A strength of the present study was evaluating the 10-time chair rise test [19], an objective evaluation of functional performance that is responsive to change and predictive of poor outcomes [20, 21]. Few prior studies have reported on how objectively quantified physical performance changes after COVID-19 hospitalization. The 6 min walk test (6-MWT) [30] is used most often, however, it measures endurance rather than strength, is limited by dyspnea, cannot be performed in many clinical locations due to space constraints, and is not easily done in a virtual setting. Huang and colleagues found that 23% of participants 6 months after COVID-19 had 6-MWT distance below the lower limit of the normal range [4]. Wu and colleagues reported significant improvements in the 6-MWT distance from 3 months to 6 months after COVID-19 in 83 participants post-hospitalization, with further improvements continuing at 9 and 12 months [31]. Arnold et al., reported conducting a 1-min chair stand test in 110 COVID-19 survivors 8–12 weeks after COVID-19 hospitalization, but results of their chair stand test do not appear in their brief communication article [5]. In the present study, 89% improved, and participants > 60 years of age tended to improve more than those under age 45, even with adjustment for the weeks 2–6 10-time chair rise. This may reflect a healthier older population that did not require a higher level of care at hospital discharge or greater drop-out among younger participants with full recovery, although the present study may select for healthier older participants as it required patients to survive their hospitalization and be discharged home (or to their prior place of residence) to be eligible for the study. Further investigation of how age and other factors may impact long-term COVID-19 recovery may help inform rehabilitation needs and referrals in the future.

The impact of COVID-19 hospitalization on change in frailty status at multiple time points is a second notable finding of our study. While a variety of factors have been implicated in morbidity and mortality [2, 32, 33], frailty may be an especially important predictor of outcomes for individuals with COVID-19 [11–14] (and other diseases and conditions). Two large meta-analyses have identified baseline frailty as a predictor of mortality from COVID-19 [11, 12]. A prospective cohort study of participants with COVID-19 over age 60 identified frailty as a significant and strong predictor of disease severity even after adjusting for age, sex, body mass index, and multiple laboratory values [13]. Two-thirds of participants aged 65 years and older who have been hospitalized with COVID-19 are frail, with higher prevalence of frailty in women compared to men and in older participants compared to younger ones [15]. We found that among those who completed the pre-hospitalization assessment, 65% reported being ‘very fit’ or ‘well’ prior to hospitalization with COVID-19. In contrast, just 21, 41, and 49% at our follow-up timepoints (2–6, 12, and 18 weeks post-discharge, respectively) reported being ‘very fit’ or ‘well’, and 67% of respondents at week 2–6 selected a worse frailty condition compared to their pre-hospitalization level. Our findings suggest new onset of pre-frailty or frailty occurs in a substantial percentage of individuals following COVID-19 hospitalization (82% of previously non-frail participants) and fails to resolve in some by 18 weeks later (39% of previously non-frail). It is possible, however, that the Clinical Frailty Scale is more sensitive to functional deterioration rather than small improvements in function and that recovery to higher functional performance is more difficult than recovering to lower levels. Ongoing frailty may have a major impact on healthcare utilization and vulnerability to subsequent infections or conditions; persons with ongoing or worsening frailty may benefit from rehabilitation programs to improve return to pre-hospital state.

We found substantially lower quality of life, evaluated using the WHODAS 2.0, in our study participants compared to normative data [25]. At 2–6 weeks after discharge, the mean score on the WHODAS 2.0 was 15, which is the 95th (worst) percentile score for healthy individuals age 45–54 years (our study mean age was 53). While scores improved on average 6.8 points, our mean scores at weeks 12 and 18 were still between the 85th and 90th percentiles for individuals aged 45–54 years, suggesting a lingering impact of COVID-19 hospitalization.

We also found that symptoms of probable PTSD were present in many individuals after COVID-19, as has been observed following intensive care unit hospitalization due to non-COVID-19 related causes [34]. Among the 85 individuals in the present study who completed the IES at least once, 46% (including 50% [11/22] who were admitted to the ICU versus 44% [28/63] who were not) met the IES threshold for probable PTSD [27]. Similarly, nearly half of all participants (238/488, or 49%) in the Michigan cohort study reported being emotionally affected by their health 60 days after being discharged from COVID-19 hospitalization [3]. In a purposive sample of 100 survivors of COVID-19 4–8 weeks after hospitalization, Halpin et al. found that 24% of participants in the hospital ward and 47% of participants in the ICU had PTSD symptoms, including 77% of females compared to just 39% of males in the ICU [6].

We found several consistent characteristics associated with lingering impairments following COVID-19 hospitalization. Participants who did not return to pre-hospitalization frailty levels were more likely to be female, were more likely to have reported pre-hospitalization frailty category of ‘very fit’ or ‘well’, and tended to be younger. Similarly, participants who did not return to their pre-hospitalization exercise level tended to be younger and have answered ‘very fit’ or ‘well’ on the pre-hospitalization frailty assessment. Interestingly, participants under age 45 years were more likely to be admitted to the ICU compared to participants aged 45–59 years and those over age 60 years. Similar to our findings, Halpin et al. reported the median age of participants admitted to the ICU was younger (58.5 years) compared to those in the hospital ward (70.5 years) [6]. Moreno-Perez found that persistent anosmia-dysgeusia was associated with younger age, although they identified no baseline clinical features (including age, sex, ICU admission, hospital/ICU length of stay, etc.) as independent predictors of PASC [8]. It is possible that older participants, including those with milder cases of COVID-19, were more likely to be hospitalized and/or more likely to go to a skilled nursing facility following hospitalization (thus excluded from the present study because of difficulty with follow-up early after hospitalization). Interestingly, participants who have higher pre-hospitalization fitness levels may have more difficulty, or at least more perceived difficulty, returning to those levels despite having better overall outcomes. Our findings suggest that females, younger participants, and those who are ‘very fit’ or ‘well’ prior to hospitalization may be less likely to perceive recovery to prior functional levels thus could be more likely to benefit from rehabilitation services. Further research is needed.

Longitudinal assessments are another strength of our study. While many studies are limited to one cross-sectional analysis, Wu and colleagues recently published a study evaluating respiratory outcomes 3, 6, 9, and 12 months following COVID-19-related hospitalization [31]. Dyspnea scores and exercise capacity improved over time in the majority of the 83 participants, but a subgroup of participants 12 months after COVID-19 hospitalization had persistent physiological and radiographic changes. Moreno-Perez also reported on select symptoms at multiple timepoints after COVID-19 hospitalization (2–3 months and 4 months), finding a lower prevalence of persistent dyspnea and cough at 4 months compared to 2–3 months after COVID-19 infection in 277 survivors [8].

There are limitations to consider when interpreting the results of the study. As discussed above, missing data was more common in younger participants and minorities, indicating our findings may not fully represent COVID-19 survivors discharged from hospital; however, we did have a strong minority representation, as 59% of participants in the present study identified as an ethnic and/or racial minority. We also only included participants who could understand English and/or Spanish, although most potential participants understood at least one of these languages. Many eligible patients were not included due to patient volume, a lack of valid contact information, failure to return calls/emails, and individuals declining enrollment. While only 109 (10.6%) of 1031 patients whom we attempted to contact participated in the study, the most common reasons for not enrolling in the study were not answering the phone or email (*n* = 428), declining to participate (*n* = 208), and phone or technology issues (*n* = 99). Only 80 (7.8%) individuals were ineligible due to medical reasons. We believe the sample accurately reflects the majority of patients hospitalized due to COVID-19 except for those in the frailest pre-hospitalization categories. Our chair rise tests (and all assessments) were conducted virtually; while we took measures to ensure accuracy (including having the participant count aloud and use video when possible), this form of data collection has not been validated though is described by The Gerontological Society of America [35]. Performing the tests remotely, however, likely allowed us to capture a greater proportion of participants, thus our findings may be more generalizable. Finally, our self-assessed clinical frailty measure may not correspond to a provider-assessed scale, thus could capture perceived changes that are important to patients.

## Conclusion

The purpose of this study was to prospectively evaluate and describe recovery in participants at multiple time-points following COVID-19 hospitalization. We found that although functional performance improved up to 18 weeks following hospitalization, functional impairments and frailty persisted in a subset of patients. Potential explanatory factors, including age, sex, race/ethnicity, and pre-hospitalization frailty status, impacted recovery from COVID-19 on some variables but not others. The results of the present study provide insight into the trajectory of recovery among an observational cohort of individuals (mean age 53 years) who were hospitalized due to COVID-19 and may inform how providers counsel patients and/or direct additional treatments or therapies.

## Supplementary Information

**Additional file 1: Appendix 1.** Patient characteristics stratified by categorical age. **Appendix 2.** Demographics stratified by sex. **Appendix 3.** Demographics stratified by race/ethnicity groups. **Appendix 4.** Counts of surveys and calls completed by weeks post-hospitalization and categorical age.

**Additional file 2: Supplemental Figure 1.** The flow chart depicts how many individuals were contacted and reasons why individuals did not enroll or participate in the study.

## Data Availability

All data generated or analyzed during this study are included in this published article [and its supplementary information files]. The datasets used and/or analyzed during the current study are available from the corresponding author on reasonable request.

## Abbreviations

PASC: Post-acute sequelae of COVID-19
ICU: Intensive Care Unit
PTSD: Post-traumatic stress disorder
SPPB: Short Physical Performance Battery
6-MWT: 6-min walk test
