# Supplementary material for: Prospective telehealth analysis of functional performance, frailty, quality of life, and mental health after COVID-19 hospitalization
Source: BMC Geriatr. 2022 Mar 26;22:251. doi: 10.1186/s12877-022-02854-6 (PMC8956362; doi:10.1186/s12877-022-02854-6)
Supplement: Supplementary file 1 — Additional file 1: Appendix 1. Patient characteristics stratified by categorical age. Appendix 2. Demographics stratified by sex. Appendix 3. Demographics stratified by race/ethnicity groups. Appendix 4. Counts of surveys and calls completed by weeks post-hospitalization and categorical age. [file 12877_2022_2854_MOESM1_ESM.docx]

**Appendix 1: Patient characteristics stratified by categorical age**

|  | ﻿ | <45 | 45-59 | 60 or greater |
| --- | --- | --- | --- | --- |
| n |  | 29 | 37 | 43 |
| Age at Admission (mean (SD)) | | 35.8 (6.8) | 51.6 (4.0) | 65.7 (5.2) |
| Male (%) |  | 11 (37.9) | 15 (40.5) | 25 (58.1) |
| Race/Ethnicity (%) | |  |  |  |
|  | Black/African-American, non-Hispanic | 5 (17.2) | 7 (18.9) | 12 (27.9) |
|  | Hispanic, regardless of race | 14 (48.3) | 16 (43.2) | 4 (9.3) |
|  | Other race, non-Hispanic | 2 (6.9) | 3 (8.1) | 1 (2.3) |
|  | White, non-Hispanic/unknown | 8 (27.6) | 11 (29.7) | 26 (60.5) |
| Education (%) | |  |  |  |
|  | Some high school | 1 (3.4) | 3 (8.1) | 1 (2.3) |
|  | High School graduate or GED | 5 (17.2) | 3 (8.1) | 4 (9.3) |
|  | Some college or associate’s degree | 8 (27.6) | 5 (13.5) | 10 (23.3) |
|  | Completed college or bachelor’s degree | 1 (3.4) | 5 (13.5) | 8 (18.6) |
|  | Post-college (regardless of degree) | 3 (10.3) | 3 (8.1) | 7 (16.3) |
|  | Missing | 11 (37.9) | 18 (48.6) | 13 (30.2) |
| Pre-hospitalization employment (%) | |  |  |  |
|  | Working, full or part time | 17 (58.6) | 13 (35.1) | 19 (44.2) |
|  | Missing | 10 (34.5) | 18 (48.6) | 11 (25.6) |
| Median days hospitalized [IQR] | | 5.0 [2.0, 8.5] | 5.0 [3.0, 9.0] | 4.0 [3.0, 7.5] |
| Admission to the intensive care unit (ICU) (%) | | 13 (44.8) | 8 (21.6) | 7 (16.3) |
| Intubated (%) | | 6 (20.7) | 3 (8.1) | 5 (11.6) |
| Discharge location (%) | |  |  |  |
|  | Home | 28 (96.6) | 34 (91.9) | 36 (83.7) |
|  | Home with home health | 0 (0.0) | 1 (2.7) | 3 (7.0) |
|  | Facility | 1 (3.4) | 0 (0.0) | 3 (7.0) |
|  | Other | 0 (0.0) | 2 (5.4) | 1 (2.3) |
| Discharged on oxygen (%) | | 17 (58.6) | 19 (51.4) | 20 (46.5) |

**Appendix 2: Demographics stratified by sex**

|  | ﻿ | Female | Male |
| --- | --- | --- | --- |
| n |  | 58 | 51 |
| Age at admission (mean (SD)) | | 51.2 (13.3) | 55.1 (12.8) |
| Categorical age (%) | |  |  |
|  | <45 | 18 (31.0) | 11 (21.6) |
|  | 45-59 | 22 (37.9) | 15 (29.4) |
|  | 60 or greater | 18 (31.0) | 25 (49.0) |
| Race/Ethnicity (%) | |  |  |
|  | Black/African-American, non-Hispanic | 12 (20.7) | 12 (23.5) |
|  | Hispanic, regardless of race | 22 (37.9) | 12 (23.5) |
|  | White, non-Hispanic | 22 (37.9) | 23 (45.1) |
|  | Other, non-Hispanic/unknown | 2 (3.4) | 4 ( 7.8) |
| Education (%) | |  |  |
|  | Some high school | 3 (5.2) | 2 (3.9) |
|  | High School graduate or GED | 8 (13.8) | 4 (7.8) |
|  | Some college or associate’s degree | 9 (15.5) | 14 (27.5) |
|  | Completed college or bachelor’s degree | 10 (17.2) | 4 (7.8) |
|  | Post-college (regardless of degree) | 5 (8.6) | 8 (15.7) |
|  | Missing | 23 (39.7) | 19 (37.3) |
| Pre-hospitalization employment (%) | |  |  |
|  | Working, full time or part time | 25 (43.1) | 24 (47.1) |
|  | Missing | 22 (37.9) | 17 (33.3) |
| Median days hospitalized [IQR] | | 5.0 [3.0, 9.0] | 4.0 [2.0, 8.0] |
| Admission to Intensive Care Unit (ICU) | | 14 (24.1) | 14 (27.5) |
| Intubated (%) | | 8 (13.8) | 6 (11.8) |
| Discharge location (%) | |  |  |
|  | Home | 53 (91.4) | 45 (88.2) |
|  | Home with home health | 2 ( 3.4) | 2 ( 3.9) |
|  | LTCF | 1 ( 1.7) | 3 ( 5.9) |
|  | Other | 2 ( 3.4) | 1 ( 2.0) |
| Discharged on oxygen = Yes (%) | | 25 (43.1) | 31 ( 60.8) |

**Appendix 3: Demographics stratified by race/ethnicity groups**

|  | ﻿ | White, non-Hispanic (ref) | Black/African-American, non-Hispanic | Hispanic, regardless of race |
| --- | --- | --- | --- | --- |
| n |  | 45 | 24 | 34 |
| Age at admission (mean (SD)) | | 57.7 (12.9) | 54.8 (11.4) | 46.0 (12.2) |
| Categorical age (%) | |  |  |  |
|  | <45 | 8 (17.8) | 5 (20.8) | 14 (41.2) |
|  | 45-59 | 11 (24.4) | 7 (29.2) | 16 (47.1) |
|  | 60 or greater | 26 (57.8) | 12 (50.0) | 4 (11.8) |
| Male | (%) | 23 (51.1) | 12 (50.0) | 12 ( 35.3) |
| Education (%) | |  |  |  |
|  | Some high school | 0 (0.0) | 1 (4.2) | 4 (11.8) |
|  | High School graduate or GED | 4 (8.9) | 3 (12.5) | 5 (14.7) |
|  | Some college or associate’s degree | 12 (26.7) | 7 (29.2) | 2 ( 5.9) |
|  | Completed college or bachelor’s degree | 11 (24.4) | 0 (0.0) | 1 (2.9) |
|  | Post-college (regardless of degree) | 8 (17.8) | 2 ( 8.3) | 2 (5.9) |
|  | Missing | 10 (22.2) | 11 (45.8) | 20 (58.8) |
| Pre-hospitalization full- or part-time employment (%) | | |  |  |
|  | No | 13 (28.9) | 3 (12.5) | 3 (8.8) |
|  | Yes | 22 (48.9) | 12 (50.0) | 12 (35.3) |
|  | Missing | 10 (22.2) | 9 (37.5) | 19 (55.9) |
| Median days hospitalized [IQR] | | 4.0 [3.0, 7.0] | 5.0 [3.8, 12.8] | 4.0 [2.0, 10.0] |
| Admission to the intensive care unit (ICU) (%) | | 10 (22.2) | 8 ( 33.3) | 10 (29.4) |
| Intubated (%) | | 5 (11.1) | 5 ( 20.8) | 4 (11.8) |
| Discharge location (%) | |  |  |  |
|  | Home | 41 (91.1) | 19 ( 79.2) | 32 (94.1) |
|  | Home with home health | 1 (2.2) | 3 (12.5) | 0 (0.0) |
|  | Facility | 2 (4.4) | 1 (4.2) | 1 (2.9) |
|  | Other | 1 (2.2) | 1 (4.2) | 1 (2.9) |
| Discharged on oxygen (%) | | 23 (51.5) | 12 (50.0) | 17 (50.0) |

**Appendix 4.** Counts of surveys and calls completed by weeks post-hospitalization and categorical age.

|  | **Surveys Completed** | | | | **Interview Calls Completed** | | | | **Completed Both** |
| --- | --- | --- | --- | --- | --- | --- | --- | --- | --- |
| **Categorical Age** | **< 45** | **45-59** | **≥ 60** | **Total** | **< 45** | **45-59** | **≥ 60** | **Total** | **Total** |
| **N** | 29 | 37 | 43 | **109** | 29 | 37 | 43 | **109** | **109** |
| Weeks 2-6 (%) | 19 (59) | 19 (48) | 31 (70) | **69 (59)** | 24 (75) | 36 (90) | 41 (93) | **101 (87)** | **68 (62)** |
| Week 12 (%) | 12 (38) | 16 (40) | 26 (59) | **54 (47)** | 11 (34) | 25 (63) | 31 (70) | **67 (58)** | **42 (39)** |
| Week 18 (%) | 13 (41) | 20 (50) | 25 (57) | **58 (50)** | 12 (38) | 20 (50) | 31 (70) | **63 (54)** | **43 (39)** |
| Completed all 3 (%) | 7 (22) | 12 (30) | 18 (41) | **37 (32)** | 7 (22) | 18 (45) | 28 (64) | **53 (46)** | **24 (22)** |
| Completed the first, and week 12 or 18 (%) | 12 (38) | 16 (40) | 27 (61) | **55 (47)** | 10 (31) | 26 (65) | 32 (73) | **68 (59)** | **43 (39)** |
