# Supplementary material for: Prospective telehealth analysis of functional performance, frailty, quality of life, and mental health after COVID-19 hospitalization
Source: BMC Geriatr. 2022 Mar 26;22:251. doi: 10.1186/s12877-022-02854-6 (PMC8956362; doi:10.1186/s12877-022-02854-6)

**Supplemental Figure 1.** The flow chart depicts how many individuals were contacted and reasons why individuals did not enroll or participate in the study.


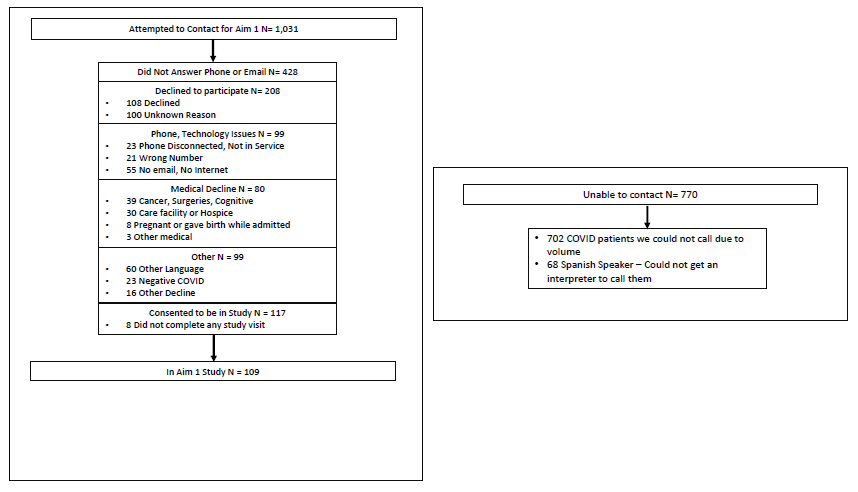

Supplement: Supplementary file 2 — Additional file 2: Supplemental Figure 1. The flow chart depicts how many individuals were contacted and reasons why individuals did not enroll or participate in the study. [file 12877_2022_2854_MOESM2_ESM.docx]
